# Memory suppression trades prolonged fear and sleep-dependent fear plasticity for the avoidance of current fear

**DOI:** 10.1038/srep02227

**Published:** 2013-07-18

**Authors:** Kenichi Kuriyama, Motoyasu Honma, Takuya Yoshiike, Yoshiharu Kim

**Affiliations:** 1 Department of Adult Mental Health, National Institute of Mental Health, National Center of Neurology and Psychiatry, Japan

## Abstract

Sleep deprivation immediately following an aversive event reduces fear by preventing memory consolidation during homeostatic sleep. This suggests that acute insomnia might act prophylactically against the development of posttraumatic stress disorder (PTSD) even though it is also a possible risk factor for PTSD. We examined total sleep deprivation and memory suppression to evaluate the effects of these interventions on subsequent aversive memory formation and fear conditioning. Active suppression of aversive memory impaired retention of event memory. However, although the remembered fear was more reduced in sleep-deprived than sleep-control subjects, suppressed fear increased, and seemed to abandon the sleep-dependent plasticity of fear. Active memory suppression, which provides a psychological model for Freud's ego defense mechanism, enhances fear and casts doubt on the potential of acute insomnia as a prophylactic measure against PTSD. Our findings bring into question the role of sleep in aversive-memory consolidation in clinical PTSD pathophysiology.

Aversive experiences (e.g., those encountered during combat, crime, abuse, disaster, and motor vehicle accidents [MVAs]) can facilitate the development of posttraumatic stress disorder (PTSD). Specifically, long-term memory consolidation of these aversive experiences is thought to be involved in the pathology of PTSD^1^,^2^. Sleep is known to play a crucial role in memory consolidation, especially in regard to fear associations^3^,^4^. When sleep is prevented immediately following an aversive event, fear associations are not formed because normal memory consolidation during homeostatic sleep is prevented^5^,^6^,^7^. Therefore, although acute insomnia may be a plausible risk factor for PTSD^8^, it can act prophylactically against the development of PTSD in traumatized patients.

Retention of event-related memory is impaired by the active suppression of traumatic memory acquisition or retrieval during or immediately after a traumatic experience^9^.This suppression provides a psychological model for Freud's proposal that repression is an ego defense mechanism^10^. From a clinical perspective, dissociation, which may involve suppression of the overwhelming emotional content of the aversive experience in the aftermath of trauma, is a common feature of PTSD^11^. Suppressed (or repressed) aversive memories could cause pathological fear when the aversive experience is recognized. To examine the suppression of unwanted aversive memories, we used an adaptation of the directed forgetting (DF) paradigm^12^,^13^ in a contextual event-emotion association memory task^7^,^14^. The DF paradigm involves active suppression of memory acquisition or retrieval immediately after a mnemonic item-pair presentation: subjects are presented with target word pairs and instructed to forget the pairs. This paradigm can impair the retention of declarative memory associations by selectively suppressing recognition performance when recalling specific word pair associations^12^,^13^. Active suppression by DF promotes prefrontal activation and reduces hippocampal activation during encoding and recollection^15^. Because hippocampal activation indexes successful recollection^16^, low activation in the hippocampus correlates with memory recollection impairment^15^. As the hippocampus contributes to encoding associations between declarative information rather than contextual fear associations^17^, the DF paradigm can selectively deteriorate the accuracy of contextual recognition of events, although it may not be able to suppress the contextual associations between facts and the fear emotion.

Moreover, an interaction between DF and sleep on memory processing has been reported^18^,^19^. Sleep followed by DF facilitates memory damping; greater recognition accuracy was achieved in mnemonic items to be remembered than those to be forgotten. Even though there have been clinical signs of a possible interaction between active suppression of aversive memories and sleep deprivation, no basic evidence yet exists to support such an interaction. This study sought to elucidate the involvement of sleep in the suppression of aversive event memory acquisition, in order to better understand whether acute insomnia following trauma constitutes a risk for or protection against PTSD.

## Results

### Sleep duration and vigilance level

**Figure 1:**
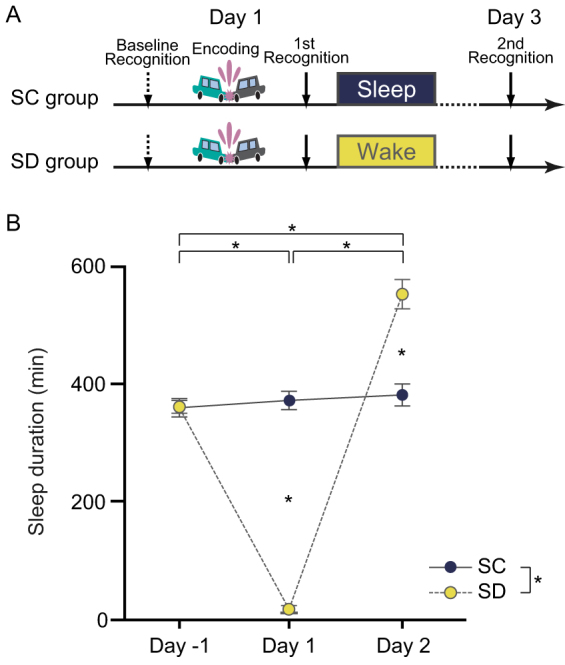
Experimental protocol and Inter-group differences in the duration of sleep. (A) Experimental sessions were conducted on Days 1 and 3 as part of a 3-day protocol. Subjects completed the baseline and encoding trials between 16:00 and 16:30 and the first recognition trial between 16:30 and 17:00 on the same day (Day 1), and the second recognition trial between 16:30 and 17:00 on Day 3. Subjects were randomly assigned to the total sleep deprivation (SD) or sleep control (SC) groups and then further randomly divided into 2 subgroups, directed forgetting (DF) or directed remembering (DR). (B) SC subjects slept for approximately 6 h per night (371.3 ± 9.49 min) throughout the experiment. SD subjects were deprived of all sleep on the first experimental night (14.94 ± 5.69 min), and the second night of sleep was prolonged for homeostatic recovery by about 2–3 h more than the usual sleep length (553.3 ± 24.0 min). Bars and error bars represent mean and standard error of the mean (SEM), respectively. *p < 0.0001.

Sixty-two healthy human subjects were randomly assigned to a total sleep deprivation (SD) or sleep control (SC) group and then further randomly divided into directed forgetting (DF) or directed remembering (DR) subgroups. They took part in the 3-day memory experiment (Fig. 1A). After Day 1, SD subjects (*n* = 31) were totally deprived of nocturnal sleep and obtained sufficient recovery sleep on Day 2 (Fig. 1B), while SC subjects (*n* = 31) were allowed to sleep normally throughout the experimental period. Sleep duration on Day 1 was significantly shorter than on Day -1, *Δ* [difference between mean values] = −167.0 min, *p* < 0.0001, and Day 2, *Δ* = −274.2 min, *p* < 0.0001, and sleep duration on Day 2 was significantly longer than on Day -1, *Δ* = 107.2 min, *p* < 0.0001 (all post-hoc tests). These statistical detections were guaranteed by significant day (F_2,174_ = 146.7, *p* < 0.0001) and sleep (F_1,174_ = 21.56, *p* < 0.0001) effects and a sleep × day interaction (F_2,174_ = 138.8, *p* < 0.0001) related to sleep duration in a precedent 3-way (sleep × encoding × day) analysis of variance (ANOVA). SC subjects had significantly more sleep during the study (*Δ* = 61.67 min, *p* < 0.0001; post-hoc test) than SD subjects. Sleep duration for SD subjects was significantly shorter on Day 1 (*Δ* = −356.9 min, *t*_60_ = 20.64, *p* < 0.0001) and significantly longer on Day 2 (*Δ* = 171.3 min, *t*_60_ = −5.71, *p* < 0.0001) than for SC subjects (follow-up *t-*tests; Fig. 1B).

Each day, the Stanford Sleepiness Scale (SSS) was used to estimate the subjective vigilance levels of all subjects at the beginning of the experimental session^20^. The SSS revealed no differences in vigilance levels between subjects regardless of sleep condition (*p* = 0.598), encoding condition (*p* = 0.823), recognition session (*p* = 0.993), or any potential interactions (*ps* > 0.1; 3-way [sleep × encoding × day] ANOVA).

### Immediate emotional reaction to an aversive event

**Figure 2:**
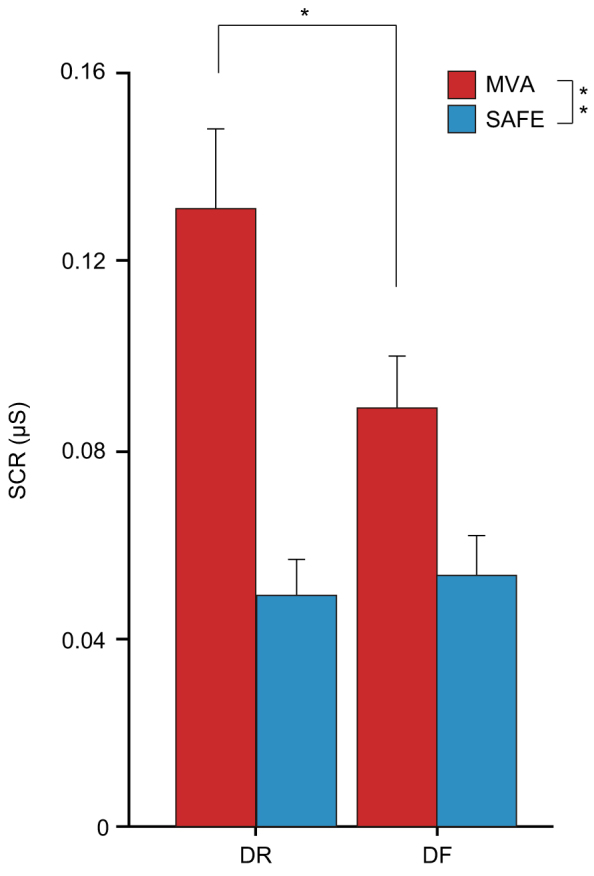
Emotional response determined by skin conductance response (SCR) during the viewing of movie clips during encoding. Subjects showed a stronger emotional stress response to movie clips of a motor vehicle accident (MVA) than to those of SAFE driving (*Δ* = 0.058 μS), regardless of sleep condition. DR subjects showed greater emotional stress response to MVA movies than DF subjects (*Δ* = 0.042 μS). Bars and error bars represent mean and standard error of the mean (SEM), respectively. *p < 0.05, **p < 0.0001.

A 3-way (context × sleep × encoding) ANOVA showed a significant main effect of context (F_1,116_ = 26.77, *p* < 0.0001) and a significant context × encoding interaction (F_1,116_ = 4.26, *p* = 0.0412) on skin conductance response (SCR) measurements. SCR was greater when viewing motor vehicle accident (MVA) movies than when viewing safe driving situation (SAFE) movies (*p* < 0.0001). In addition, in the MVA context, SCR was greater in DR subjects than in DF subjects, regardless of sleep condition (Fig. 2). Follow-up *t*-tests showed a significant difference in SCR between DF and DR subjects while watching MVA movies (*t*_60_ = −2.18, *p* = 0.0334). The results suggest that DF successfully reduced the emotional stress response to an aversive event.

### Explicit contextual recognition of an event

**Figure 3:**
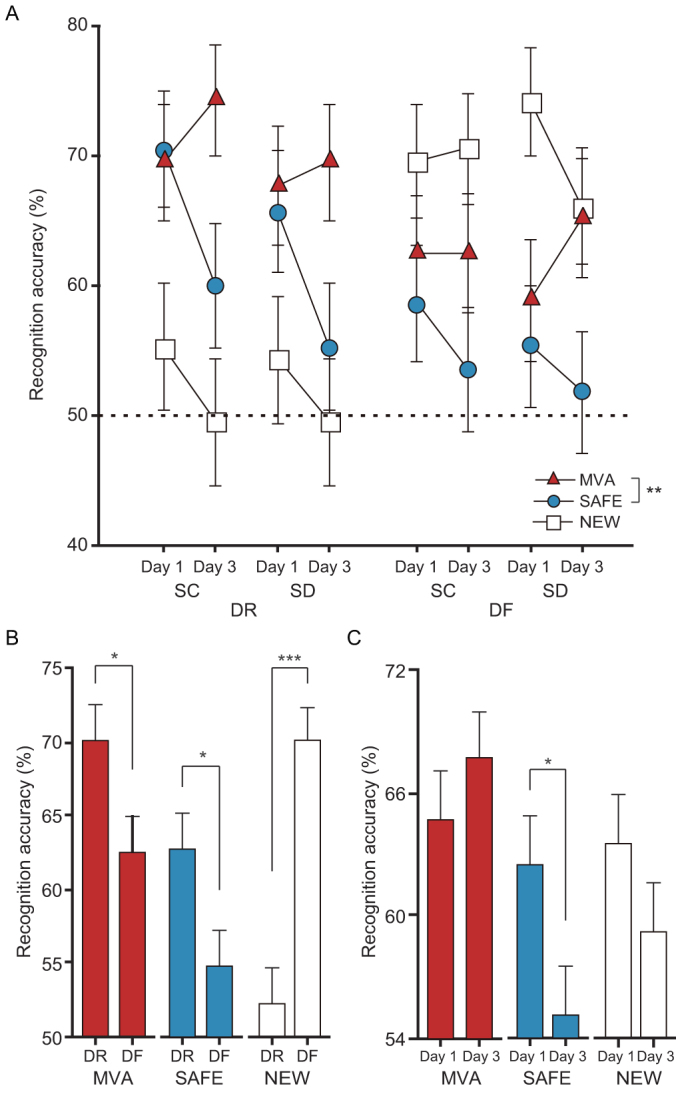
Factorial contribution to recognition accuracy during event recognition trials, and subsequent analyses of context × encoding and context × day interaction. (A) Greater recognition accuracy was seen in the MVA context than in the SAFE context (*Δ* = 7.5%). (B) DF greatly enhanced recognition accuracy in the NEW context (*Δ* = 17.9%) but reduced recognition accuracy in the MVA (*Δ* = −7.7%) and SAFE (*Δ* = −7.9%) contexts. (C) Recognition accuracy in the SAFE context was reduced on Day 3 (*Δ* = 7.4%), but accuracies in the MVA and NEW contexts were retained until Day 3. Data points (or bars) and error bars represent mean and SEM, respectively. *p < 0.05, **p < 0.005, ***p < 0.0001.

For recognition accuracy, a 4-way ANOVA (context × sleep × encoding × day) showed a significant context effect (F_2,2580_ = 5.53, *p* = 0.004) and a significant context × encoding interaction (F_2,2580_ = 20.75, *p* < 0.0001), and a trend toward significance in the context × day interaction (F_2,2580_ = 2.72, *p* = 0.066: Fig. 3A and Supplemental Table 1). Sleep deprivation had no effect on event recognition accuracy regardless of encoding condition or recognition context. Post-hoc tests showed a significant difference between the MVA and SAFE contexts in recognition accuracy (*Δ* = 7.5%, *p* = 0.0012). DF reduced recognition accuracy of previously seen (OLD) events (the MVA and SAFE contexts), whereas it improved accuracy for never-before-seen (NEW) events. Follow-up *t*-tests revealed that DF subjects had significantly higher accuracy in the NEW context than DR subjects (*t*_866_ = 5.52, *p* < 0.0001). By contrast, DR subjects had significantly higher accuracy in the MVA (*t*_866_ = −2.42, *p* = 0.016) and SAFE (*t*_866_ = −2.38, *p* = 0.017) contexts (Fig. 3B). DF appeared to reduce recognition accuracy for previously seen events but may also have assisted subconscious remembering that improved discrimination accuracy of never-before-seen events from previously seen events. In addition, event recognition accuracy in the MVA and NEW contexts remained high on Days 1 and 3, but in the SAFE context it had decreased by Day 3 (*t*_866_ = 2.21, *p* = 0.027: Fig. 3C). However, a non-significant difference in accuracy was seen in the MVA (*p* = 0.351) and NEW (*p* = 0.186) contexts between Days 1 and 3.

### Discriminability (d′) and recognition bias (C) in event recognition

**Figure 4:**
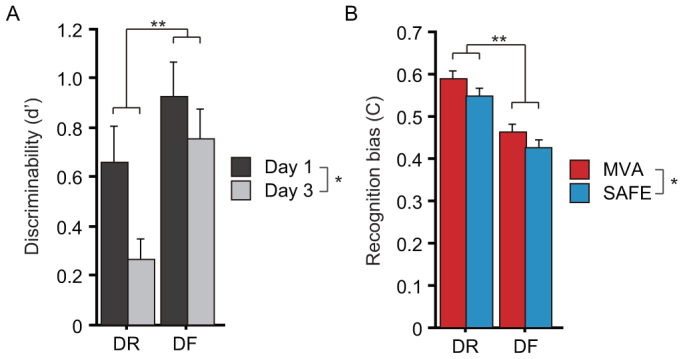
Discriminability (d′) in the event recognition task for Days 1 and 3, and recognition bias (C) in the event recognition task for MVA and SAFE events. (A) Greater discriminability was observed in the DF group than the DR group, and discriminability clearly diminished over time regardless of other factors, including sleep condition and recognition context. (B) Greater recognition bias was observed in the DR group than the DF group, and greater recognition bias was observed in the MVA context than the SAFE context regardless of the other factors, including day and sleep condition. Data points and error bars represent mean and standard error of the mean (SEM), respectively. *p < 0.05, **p < 0.005.

Four-way ANOVAs (context × sleep × encoding × day) showed significant encoding (F_1,232_ = 8.78, *p* = 0.003) and day (F_1,232_ = 4.93, *p* = 0.027) effects in discriminability (d′), and significant encoding (F_1,232_ = 44.1, *p* < 0.0001) and context (F_1,232_ = 4.30, *p* = 0.039) effects in recognition bias (C) (Supplemental Table 2). Post-hoc tests showed significantly greater discriminability in the DF subjects than the DR subjects (*Δ* = 0.38, *p* = 0.003), and significantly greater discriminability on Day 1 than on Day 3 (*Δ* = 0.28, *p* = 0.026: Fig. 4A). Post-hoc tests also showed significantly greater recognition bias in the DR subjects than the DF subjects (*Δ* = 0.12, *p* < 0.0001), and significantly greater recognition bias in the MVA context than the SAFE context (*Δ* = 0.04, *p* = 0.036: Fig. 4B). DF clearly enhanced the discrimination accuracy between NEW and OLD events and attenuated a potential memory bias. The discrimination accuracy generally diminished over time. Recognition performance in the MVA context was influenced by greater memory bias than in the SAFE context.

### Implicit emotional reaction to contextual cues

**Figure 5:**
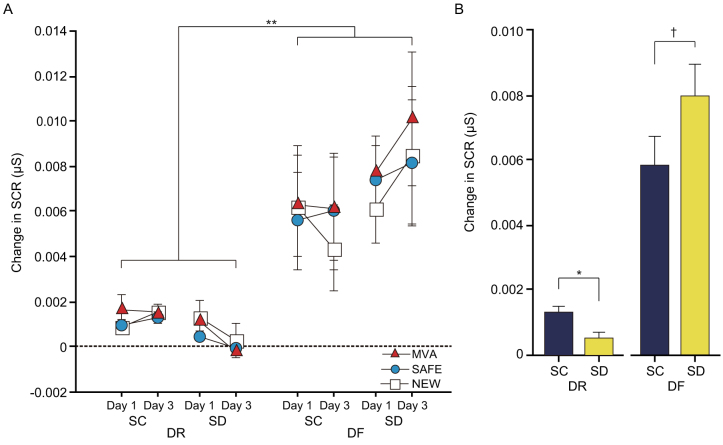
Factorial contribution to SCR during emotion recognition trials, and subsequent analysis of encoding × sleep interaction. (A) Significantly enhanced SCR was observed in all contexts on Days 1 and 3 in the DF subjects compared with the DR subjects (*Δ* = 0.006). (B) Sleep deprivation diminished SCR in all contexts for DR subjects (*Δ* = − 0.001 μS) but enhanced SCR in all contexts for DF subjects (*Δ* = 0.002 μS). The dashed line indicates SCR at baseline. Data points (or bars) and error bars represent mean and standard error of the mean (SEM), respectively. †*p* < 0.1, *p < 0.001, **p < 0.0001.

A 5-way ANOVA (context × sleep × encoding × day × correctness) showed a significant encoding effect (F_1,2556_ = 65.6, *p* < 0.0001) and a significant encoding × sleep interaction (F_1,2556_ = 4.25, *p* = 0.039) for SCR. SCR was significantly increased in the DF subjects (0.007 ± 0.001 μS) compared to the DR subjects (0.001 ± 0.0001 μS, *p* < 0.0001: Fig. 5A and Supplemental Table 3). No difference in SCR was seen between the contexts and there were no differences in relation to correct responses in the event recognition trials. This suggests that the emotional response induced by the MVA movies might manifest as a generalized emotional response in the SAFE and NEW contexts. Thus, the association between event and emotion might be blurred. Sleep deprivation dramatically reduced the SCR of DR subjects in all contexts. By contrast, in DF subjects the SCR was high in all contexts and sleep deprivation tended to enhance SCR in all contexts (Fig. 5B). Follow-up *t*-tests showed a significant inter-sleep difference in SCR for DR subjects (SC > SD, *t*_1261_ = 3.43, *p* = 0.0006) but only a trend toward significance in inter-sleep differences for DF subjects (SD > SC, *t*_1339_ = 1.63, *p* = 0.094).

## Discussion

Our results are the first to show that active memory suppression can immediately reduce the emotion-based stress reaction arising from aversive events. Further, active memory suppression appears to enhance the subsequent emotional reaction conditioned as a contextual cue associated with the aversive event; this generalized effect was observed homogeneously in all contexts regardless of memory encoding strategies due to the greatest difficulty distinguishing MVA (harmful) from SAFE (nonharmful) stimuli as well as OLD from NEW stimuli^21^,^22^.

The current results clearly indicate that the DF memory suppression strategy potentially enhanced encoding ability in the MVA and SAFE contexts (reflected in the increased number of correct rejections) independent of response bias toward suppression of recognition performance (reflected in the increased “No” responses). Anderson et al.^15^ suggested that the prefrontal activity induced by memory suppression keeps unwanted memories outside awareness, and that memory suppression interferes with recollection rather than encoding processes. Active suppression effects may also selectively persist into recognition performance; thus, recognition accuracy was reduced in the suppressed contexts in the MVA and SAFE events. By contrast, recognition accuracy was potentially enhanced in the unsuppressed context of NEW events by increased discrimination ability. This result is in line with suggestions made by Nowicka et al^23^. that a lower suppression rate for the recognition of emotionally negative pictures than for neutral ones, and the suppression of a negative emotional memory requires a greater and more widely distributed cortical network than that required for the suppression of a neutral memory.

Memories associated with a negative emotion are strongly encoded and more easily recollected than those that do not have a particularly strong associated emotion^14^,^24^,^25^. Event memory in the MVA context may have been more strongly encoded than that in the SAFE context. Thus, whereas recognition accuracy in the SAFE context decreased by Day 3, it remained high in the MVA context. Bäuml and Kuhbandner^26^ also reported that positive emotional valence restores recognition performance and eliminates the DF effect more than negative emotional valence. This would suggest that the DF effect that persisted during recognition performance could possibly be overcome by using stimuli that evoke positive rather than negative or neutral emotions, because positive emotion generates a greater motivation to remember, whereas negative emotion tends to generate a greater motivation to suppress memories.

Our results agree in part with Freud's traditional theory of hysteria (i.e., dissociative or somatoform disorders)^10^, in which he postulated that repressed, unconscious memories of trauma, such as childhood maltreatment, underlie symptom formation. Most survivors have strong memories of the traumatic event, because when a high level of arousal is experienced during such an event, one tends to focus on the central features of the event at the expense of peripheral features. However, some trauma survivors are unable to recall the most damaging core episode of their traumatic experience due to a paradoxical symptom referred to as traumatic dissociative amnesia^27^, a contentious feature of PTSD^28^. Suppression of the event could reduce the terror of the actual confrontation while enhancing the generalized fear that might contribute to traumatic dissociative amnesia.

Post-event sleep deprivation should diminish the level of the emotion-based physiological reaction by depriving the individual of the normal sleep-dependent consolidation processes involved in memory plasticity. However, the active suppression of memory during encoding was shown in the current study to disrupt this sleep deprivation effect. Instead, suppression enhanced the emotional reaction to subsequent presentation of recognition cues related to the aversive event. This gives rise to the question of why sleep deprivation did not act to deteriorate suppressed aversive events via a lack of memory consolidation of the associated emotion. Recent studies have suggested that intentionally suppressed memories of word pair associations were enhanced less by sleep than memory-directed remembering^18^,^19^. Rauchs et al.^29^ demonstrated that hippocampal activity during encoding crucially determined off-line processing during post-learning sleep and the recognition accuracy of declarative associative memory. DF attenuates hippocampal activity during encoding; therefore, suppressed memories are less enhanced by sleep^30^. Our previous study, using the same aversive stimuli, revealed that explicit event memory (of the facts) is consolidated independently from sleep-specific processing^7^. Likewise, the present results are in line with previous human and animal studies suggesting that post-event sleep deprivation attenuates the emotional response caused by an aversive event the previous day through impairment of the memory consolidation that occurs during sleep^7^,^31^.

However, although post-event sleep was prevented in the current study, the delayed emotional response was not attenuated but rather enhanced. In terms of the process of sleep-dependent memory plasticity, this is a controversial finding^32^. Memory formation of aversive experience is processed not only by the hippocampus-dominant plastic structure but also by the amygdala-dominant limbic structure^32^,^33^,^34^. When an emotion-event association is formed, these structures cooperate via certain hub structures such as the dorsal anterior cingulate cortex and the insula^24^,^35^. Memory suppression strategies could simultaneously attenuate not only hippocampal activity but also the activities of such hub structures; consequently, amygdala activity during the encoding of emotion-related information might be potentially enhanced by decreased prefrontal governance. Thus, although memory suppression appeared to instantaneously attenuate amygdala activity during the viewing of the movie clips, it actually seems to have deranged and enhanced off-line processing and the delayed recognition of emotion-based memory associations. This may be due to the incomplete establishment of contextual emotion-event associations. It has also been suggested that the different sorts of off-line processing of emotional information continue during both post-learning sleep and waking^36^. If, as proposed by Crick and Mitchinson^37^, sleep also contributes to the forgetting of irrelevant memory traces, then it is also plausible that the erasing of irrelevant information was not successful for off-line processing during post-event sleep.

The DF strategy has been most often applied to highlighting intra-individual variability in memory impairment that arises from an instruction presented before or after the presentation of unwanted material^15^. However, we applied DF to an inter-group trial, anticipating that there would be a widespread impact on memory performance due to the generalization of emotions arising from exposure to aversive stimuli. Thus, care is required when evaluating the homogeneity of these results. Furthermore, the use of both DF and SD as active interventions is a major limitation. In particular, total sleep deprivation may not be equivalent to acute stress-induced insomnia in both qualitative and quantitative aspects^38^. Further, we examined only a few days of healthy participants' recognition performance; thus, our results may provide a psychophysiological model for acute stress reactions but not generalize to acute or posttraumatic stress. There may be clinical value in disentangling the relationship between sleep disturbances, mnemonic manipulations, and posttraumatic stress symptomatology, using clinical observation. To resolve the clinical questions regarding the pathogenicity of acute stress-induced insomnia and the psychodynamic aspects of repression in the development of PTSD, a well-designed epidemiological study and a longitudinal trial that intervenes with traumatized patients in the acute stage is also required (due to specific passive concepts). Despite such potential limitations, our results offer insight into the pathogenesis of acute stress disorder and PTSD, where the psychodynamic phenomenon of repression is speculated to reflect both the nature and symptomatology of the disorders^39^.

## Methods

### Participants

Sixty-two healthy college students (28 women; mean age 21.71 [SEM ± 0.24] years, range: 20–29 years) were randomly assigned to a SD or SC group and then further randomly divided into DF or DR subgroups. This resulted in the creation of 4 groups: SD-DF (*n* = 16, 7 women; mean age 21.81 ± 0.36), SD-DR (*n* = 15, 9 women; mean age 22.20 ± 0.77), SC-DF (*n* = 16, 7 women; mean age 21.19 ± 0.25), and SC-DR (*n* = 15, 5 women; mean age 21.67 ± 0.43). The groups did not differ in mean age (F_3,58_ = 0.76, *p* = 0.52) or sex distribution (χ^2^ = 1.22, *p* = 0.75). They maintained a normal sleep-wake rhythm prior to and throughout the study other than 1 night of sleep deprivation for the SD group, at home, verified using an ambulatory wrist monitor (Actiwatch-L, Mini-Mitter Co., Inc. Bend, OR) designed to sense movement to distinguish between waking and sleeping states. The study protocol was designed in accordance with guidelines outlined in the Declaration of Helsinki and approved by the Intramural Research Board of National Center of Neurology and Psychiatry. All subjects provided written informed consent prior to participating in the study.

### Protocol

Subjects took part in the 3-day memory experiment (Fig. 1A). Immediately prior to the encoding trial and the first recognition trial on Day 1, subjects participated in a baseline trial (see Methods) to determine baseline levels for emotional responses to picture recognition evoked by the subjects' inherent arousal and affective valence. After Day 1, SD subjects (*n* = 31) were totally deprived of nocturnal sleep but obtained sufficient recovery sleep on Day 2 (Fig. 1B), while SC subjects (*n* = 31) were allowed to sleep normally throughout the experimental period. On day 3, subjects participated in the second recognition trial.Each day, the SSS was used to estimate the subjective vigilance levels of all subjects at the beginning of the experimental session^20^.

### Memory encoding task

During the encoding task^7^,^14^, subjects were required to watch fourteen 10-s movie clips. Seven of these showed a SAFE and 7 showed a MVA accompanied by real sounds heard through headphones. The clips were presented on a 28-inch LCD monitor with 30-s inter-stimulus intervals (ISIs) in randomized order. All movies were recorded via dashboard cameras built into Tokyo city cabs and showed the driver's viewpoint of a typical city street. A sudden, grisly crash involving an oncoming or crossing car, bicycle, or pedestrian appeared without warning in the second half of each MVA movie. In the SAFE movies, no remarkable event occurred. A cognitive (auditory go/no go) task was performed during the ISIs to separate the 2 consecutive stimuli. DF subjects were instructed to quickly forget what they had seen in the movie clips, despite maintaining intent gaze on the clips. DR subjects were instructed to remember what they had seen. To prevent subjects from using simple strategies to avoid stimuli exposure, such as not watching the movie clips attentively or closing their eyes, participants were monitored throughout the encoding task by a remote video camera located on the LCD monitor.

During encoding, SCR was measured in microSiemens [μS], a unit of electrical conductance^40^. Two Ag/AgCl electrodes were attached to the palmar surface of the middle and ring fingers of the non-dominant hand to estimate the stress response to the stimuli. SCR was calculated for each trial by subtracting the mean value during the ISI from the peak value during the stimulus presentation.

### Memory recognition task

An event recognition task and an emotion recognition task were used to estimate explicit declarative memory performance and implicit emotional memory performance, respectively. Accordingly, the recognition trials (still picture recognition) were run twice (for the event and emotion recognitions, respectively) per day on Days 1 and 3. Each trial involved the presentation of the same series of 21 color picture stimuli in randomized order. Subjects were shown 14 still pictures that comprised still frames from the previously presented movies (OLD pictures; 7 MVA, 7 SAFE) and 7 still pictures from similar but never-before-seen movies (NEW pictures). All pictures were taken from the first 5 s of the movies to exclude potentially recognizable scenes involving the accidents. Each stimulus was matched in terms of overall visual complexity, brightness, and contrast. The recognition task consisted of twenty-one 14-s trial runs in which a fixation crosshair was presented (7 s) during the ISI, followed by presentation of the target picture (7 s).

During the event recognition trial, subjects responded to questions related to event recognition while viewing the stimuli. All subjects were instructed to respond honestly, regardless of whether they had previously been instructed to forget or remember details of the movie clips during encoding^12^. To prevent participants from intentionally responding up to the previous instruction contexts of DF in the recognition trial, participants were monetarily rewarded with 150 dollars^12^. Subjects indicated whether they had seen the stimulus picture during the movie clips (OLD) or whether they had never seen the picture before (NEW) by pressing a corresponding key. Recognition responses were classified into 4 types: (1) correct recognition of OLD stimuli (hits), (2) incorrect recognition of OLD stimuli (misses), (3) correct recognition of NEW stimuli (correct rejections), and (4) incorrect recognition of NEW stimuli (false alarms). Recognition accuracy was defined as the percentage of questions answered correctly (hits or correct rejections). Discriminability (d′ = z [hits] – z [false alarms]) and recognition bias (C = 0.5 × z [hits] + 0.5 × z [false alarms]) were also calculated according to signal detection theory^41^. Subjects were not given feedback about their accuracy during the experiment.

During the baseline and emotion recognition trials, subjects were required to simply view the still picture stimuli while SCR was measured. This was done to avoid contamination of SCR measurements by physiological responses arising from voluntary behaviors responding to questions accompanied by involuntary responses to fear. SCR change, indicating the physiological reaction to fear conditioned to the movie clips, was calculated by subtracting the values in the baseline session from those in the recognition sessions.

### Statistics

In order to define the main outcome results, potential confounds such as sleep duration (whether SD subjects were successfully deprived of sleep) and vigilance (whether all recognition trials were reliably accomplished) were controlled. Multifactorial ANOVAs were conducted with 2 between-subjects factors (sleep [SC or SD group] and encoding [DF or DR group]) and 3 within-subjects factors (day [recognition trial conducted on Day 1 or 3], context [stimulus in the MVA, SAFE, or NEW context], and correctness [item correctly recognized or not in the explicit event recognition trial]) to analyze SCR during encoding, and event recognition accuracy, discriminability (d′), recognition bias (C), and SCR change during recognition. All ANOVAs were followed by post-hoc *t*-tests using Bonferroni correction. The significance threshold was set at 0.05 or the corresponding Bonferroni-adjusted *p* value.

## Author Contributions

K.K. designed the study and wrote the main manuscript text. M.H., T.Y., S.K. and M.K. performed the experiments. Y.K. contributed to random allocation of participants. K.K. and M.H. carried out statistical analyses. All authors contributed to the final form.

## Supplementary Material

Supplementary Information Supplementary tables
