# Supplementary material for: Memory suppression trades prolonged fear and sleep-dependent fear plasticity for the avoidance of current fear
Source: Sci Rep. 2013 Jul 18;3:2227. doi: 10.1038/srep02227 (PMC3714646; doi:10.1038/srep02227)
Supplement: Supplementary Information — Supplementary tables [file srep02227-s1.doc]

Supplemental Table 1

*Statistical Analyses of Recognition Accuracy*

| Four-way ANOVA | | |  |  | |  |  | | |  |  | |
| --- | --- | --- | --- | --- | --- | --- | --- | --- | --- | --- | --- | --- |
| Significant main effect or interaction | | |  | df | |  | F | | |  | *p* | |
| Context (MVA vs. SAFE vs. NEW) | | |  | ***2*** | |  | ***5.53*** | | |  | ***0.004*** | |
| Sleep (SC vs. SD) | | |  | 1 | |  | 1.15 | | |  | 0.284 | |
| Encoding (DF vs. DR) | | |  | 1 | |  | 0.16 | | |  | 0.690 | |
| Day (Day 1 vs. Day 3) | | |  | 1 | |  | 2.45 | | |  | 0.117 | |
| Context × Sleep | | |  | 2 | |  | 0.28 | | |  | 0.753 | |
| Context × Encoding | | |  | ***2*** | |  | ***20.75*** | | |  | ***< 0.0001*** | |
| Context × Day | | |  | *2* | |  | *2.72* | | |  | *0.066* | |
| Sleep × Encoding | | |  | 1 | |  | 0.20 | | |  | 0.659 | |
| Sleep × Day | | |  | 1 | |  | 0.01 | | |  | 0.933 | |
| Encoding × Day | | |  | 1 | |  | 0.39 | | |  | 0.535 | |
| Context × Sleep × Encoding | | |  | 2 | |  | 0.03 | | |  | 0.975 | |
| Context × Sleep × Day | | |  | 2 | |  | 0.25 | | |  | 0.782 | |
| Context × Encoding × Day | | |  | 2 | |  | 0.27 | | |  | 0.765 | |
| Sleep × Encoding × Day | | |  | 1 | |  | 0.01  .01 | | |  | 0.933 | |
| Context × Sleep × Encoding × Day | | |  | 2 | |  | 0.58 | | |  | 0.558 | |
| Follow-up *t*-tests | | | | | | | | | | | | |
|  | DF vs. DR | | | | | | |  | Day 1 vs. Day 3 | | | |
| Context | *Δ* | *t* | | | *p* | | |  | *Δ* | *t* | | *p* |
| MVA | ***- 0.077*** | ***-2.42*** | | | ***0.016*** | | |  | - 0.03 | -0.93 | | 0.351 |
| SAFE | ***- 0.079*** | ***-2.38*** | | | ***0.017*** | | |  | ***0.074*** | ***2.21*** | | ***0.027*** |
| NEW | ***0.179*** | ***5.52*** | | | ***< 0.0001*** | | |  | 0.044 | 1.33 | | 0.186 |

ANOVA = analysis of variance; df = degrees of freedom; *Δ* = difference between mean values; DF = directed forgetting; DR = directed remembering; SC = sleep control; SD = sleep deprivation; MVA = motor vehicle accident movie clips; SAFE = safe driving movie clips; NEW = never-before-seen movies. Significant values are in bold.

Supplemental Table 2

*Statistical Analyses of Discriminability (dʹ) and Recognition Bias (C) in the Event Recognition Tasks*

| Four-way ANOVA of discriminability (dʹ) |  |  |  |  |  |
| --- | --- | --- | --- | --- | --- |
| Significant main effect or interaction | df |  | F |  | *p* |
| Context (MVA vs. SAFE) | 1 |  | 1.23 |  | 0.269 |
| Sleep (SC vs. SD)    ***19.25 <.0001*** | 1 |  | 0.20 |  | 0.657 |
| Encoding (DF vs. DR) | ***1*** |  | ***8.78*** |  | ***0.003*** |
| Day (Day 1 vs. Day 3) | ***1*** |  | ***4.93*** |  | ***0.027*** |
| Context × Sleep | 1 |  | 0.03 |  | 0.872 |
| Context × Encoding | 1 |  | 0.004 |  | 0.952 |
| Context × Day | 1 |  | 0.84 |  | 0.362 |
| Sleep × Encoding | 1 |  | 0.19 |  | 0.663 |
| Sleep × Day | 1 |  | 0.26 |  | 0.610 |
| Encoding × Day | 1 |  | 0.73 |  | 0.393 |
| Context × Sleep × Encoding | 1 |  | 0.02 |  | 0.894 |
| Context × Sleep × Day | 1 |  | 0.06 |  | 0.808 |
| Context × Encoding × Day | 1 |  | 0.02 |  | 0.886 |
| Sleep × Encoding × Day | 1 |  | 0.13 |  | 0.716 |
| Context × Sleep × Encoding × Day | 1 |  | 0.13 |  | 0.723 |
| Four-way ANOVA of recognition bias (C) |  |  |  |  |  |
| Significant main effect or interaction | df |  | F |  | *p* |
| Context (MVA vs. SAFE) | ***1*** |  | ***4.30*** |  | ***0.039*** |
| Sleep (SC vs. SD)    ***19.25 <.0001*** | 1 |  | 0.38 |  | 0.538 |
| Encoding (DF vs. DR) | ***1*** |  | ***44.1*** |  | ***< 0.0001*** |
| Day (Day 1 vs. Day 3) | 1 |  | 0.26 |  | 0.613 |
| Context × Sleep | 1 |  | 0.04 |  | 0.842 |
| Context × Encoding | 1 |  | 0.01 |  | 0.934 |
| Context × Day | 1 |  | 1.62 |  | 0.205 |
| Sleep × Encoding | 1 |  | 0.10 |  | 0.753 |
| Sleep × Day | 1 |  | 0.53 |  | 0.467 |
| Encoding × Day | 1 |  | 0.05 |  | 0.828 |
| Context × Sleep × Encoding | 1 |  | 0.01 |  | 0.918 |
| Context × Sleep × Day | 1 |  | 0.01 |  | 0.946 |
| Context × Encoding × Day | 1 |  | 0.09 |  | 0.760 |
| Sleep × Encoding × Day | 1 |  | 1.17 |  | 0.280 |
| Context × Sleep × Encoding × Day | 1 |  | 0.09 |  | 0.769 |

ANOVA = analysis of variance; df = degrees of freedom; DF = directed forgetting; DR = directed remembering; SC = sleep control; SD = sleep deprivation; MVA = motor vehicle accident movie clips; SAFE = safe driving movie clips. Significant values are in bold.

Supplemental Table 3

*Statistical Analyses of Changes in Skin Conductance Response*

| Five-way ANOVA |  |  |  |  |  |
| --- | --- | --- | --- | --- | --- |
| Significant main effect or interaction | df |  | F |  | *p* |
| Context (MVA vs. SAFE vs. NEW) | 2 |  | 0.21 |  | 0.813 |
| Sleep (SC vs. SD)    ***19.25 <.0001*** | 1 |  | 0.47 |  | 0.495 |
| Encoding (DF vs. DR) | ***1*** |  | ***65.6*** |  | ***< 0.0001*** |
| Day (Day 1 vs. Day 3) | 1 |  | 0.004 |  | 0.951 |
| Correctness (Hits/Correct rejections vs. Misses/False alarms) | 1 |  | 0.56 |  | 0.456 |
| Context × Sleep | 2 |  | 0.02 |  | 0.985 |
| Context × Encoding | 2 |  | 0.14 |  | 0.870 |
| Context × Day | 2 |  | 0.03 |  | 0.972 |
| Context × Correctness | 2 |  | 0.02 |  | 0.983 |
| Sleep × Encoding | ***1*** |  | ***4.25*** |  | ***0.039*** |
| Sleep × Day | 1 |  | 0.001 |  | 0.974 |
| Sleep × Correctness | 1 |  | 0.60 |  | 0.441 |
| Encoding × Day | 1 |  | 0.25 |  | 0.621 |
| Encoding × Correctness | 1 |  | 0.24 |  | 0.625 |
| Day × Correctness | 1 |  | 0.52 |  | 0.473 |
| Context × Sleep × Encoding    ***19.25 <.0001*** | 2 |  | 0.08 |  | 0.921 |
| Context × Sleep × Day | 2 |  | 0.06 |  | 0.940 |
| Context × Sleep × Correctness | 2 |  | 0.13 |  | 0.880 |
| Context × Encoding × Day | 2 |  | 0.03 |  | 0.969 |
| Context ×Encoding × Correctness | 2 |  | 0.32 |  | 0.729 |
| Context × Day × Correctness | 2 |  | 0.15 |  | 0.863 |
| Sleep × Encoding × Day | 1 |  | 1.24 |  | 0.265 |
| Sleep × Encoding × Correctness | 1 |  | 0.14 |  | 0.705 |
| Sleep × Day × Correctness | 1 |  | 1.16 |  | 0.281 |
| Encoding × Day × Correctness | 1 |  | 0.05 |  | 0.823 |
| Context × Sleep × Encoding × Day | 2 |  | 0.11 |  | 0.893 |
| Context × Sleep × Encoding × Correctness | 2 |  | 0.08 |  | 0.927 |
| Context × Encoding × Day × Correctness | 2 |  | 0.28 |  | 0.753 |
| Context × Sleep × Day × Correctness | 2 |  | 0.33 |  | 0.719 |
| Sleep × Encoding × Day × Correctness | 1 |  | 0.02 |  | 0.895 |
| Context × Sleep × Encoding × Day × Correctness | 2 |  | 0.36 |  | 0.699 |

ANOVA = analysis of variance; df = degrees of freedom; DF = directed forgetting; DR = directed remembering; SC = sleep control; SD = sleep deprivation; MVA = motor vehicle accident movie clips; SAFE = safe driving movie clips; NEW = never-before-seen movies. Significant values are in bold.
